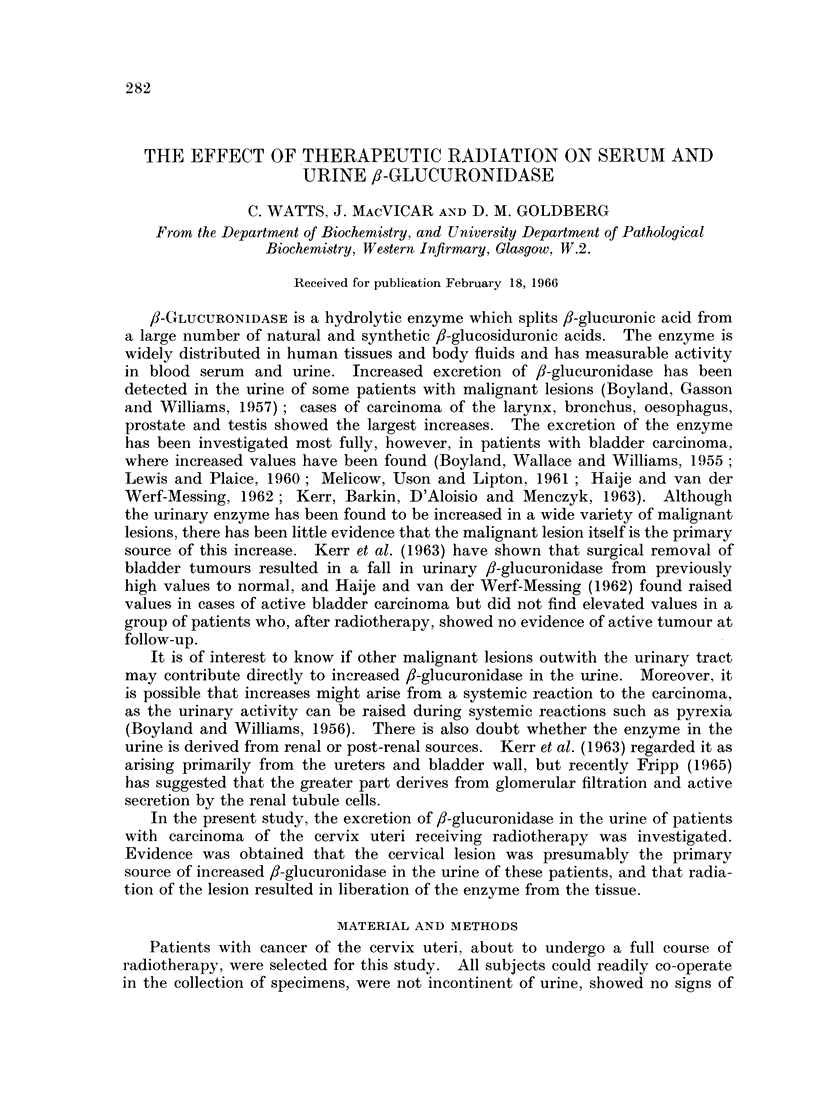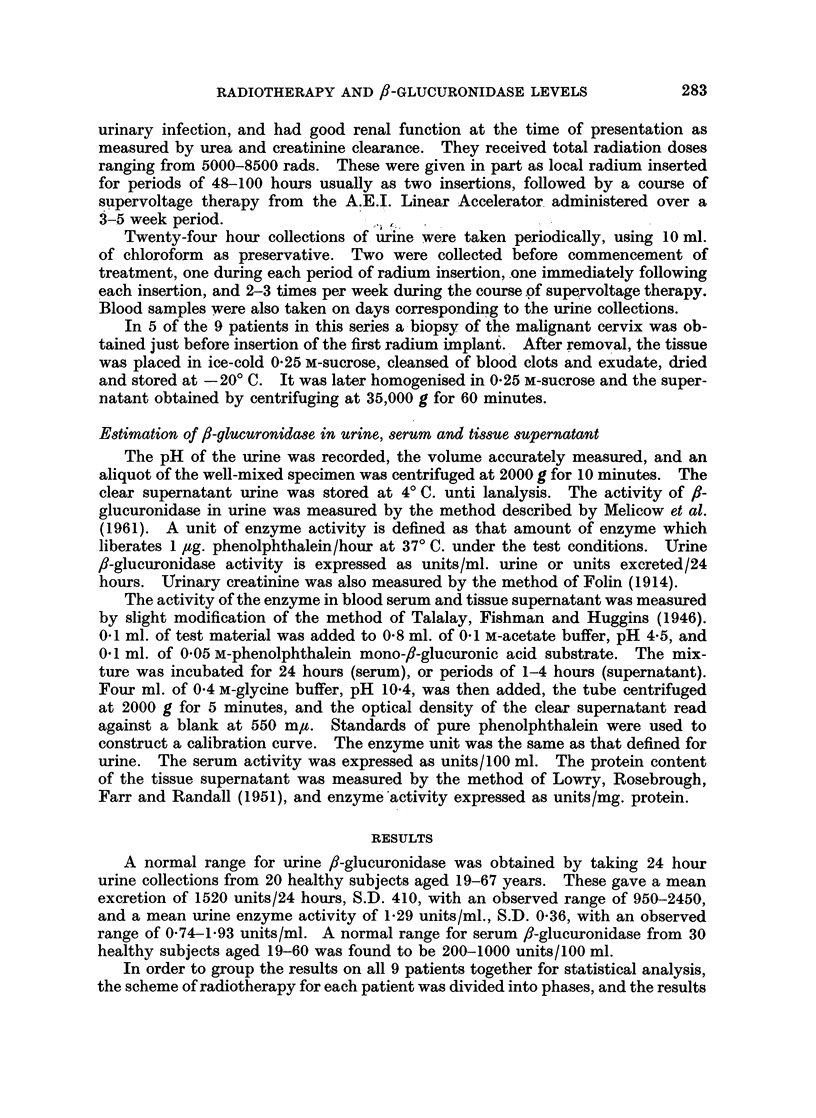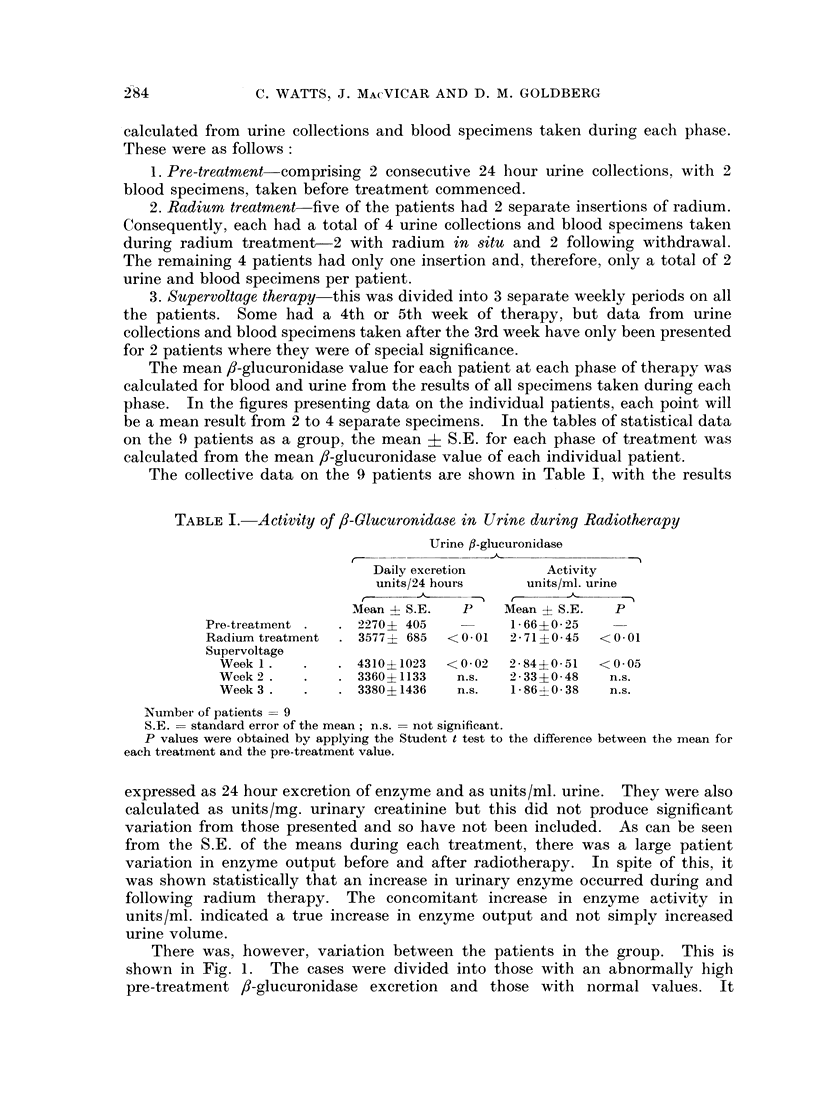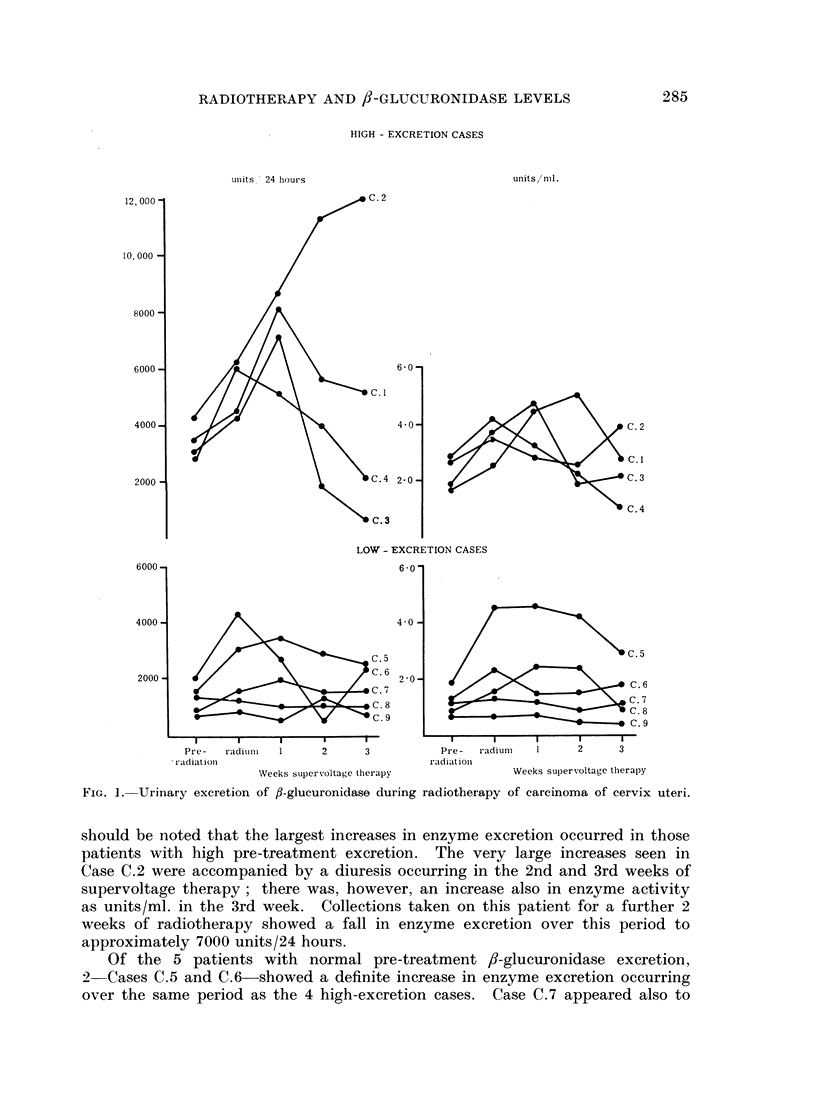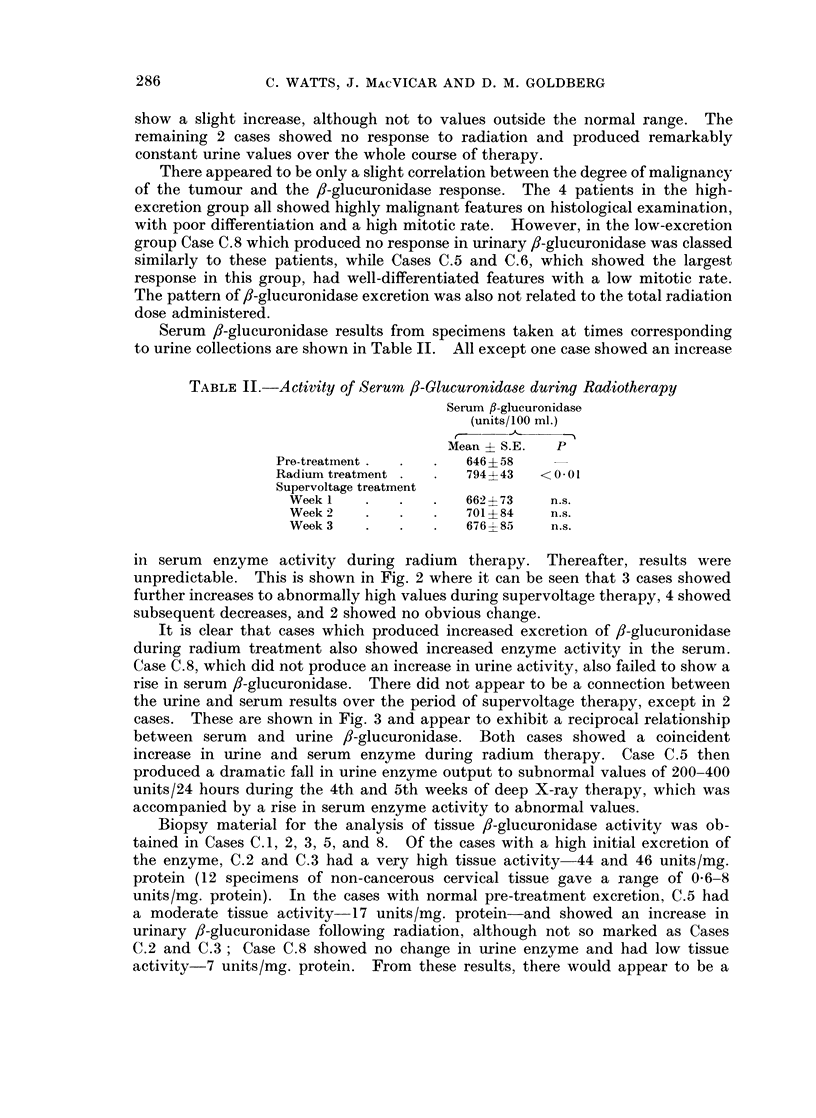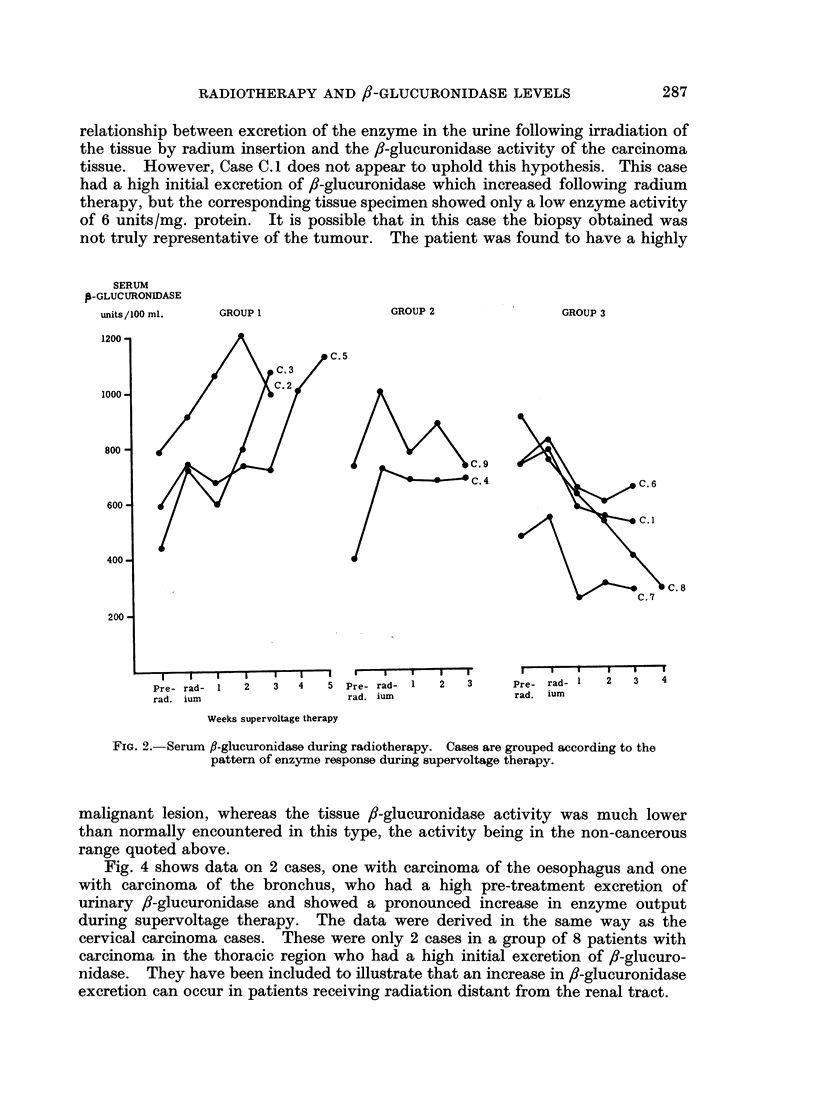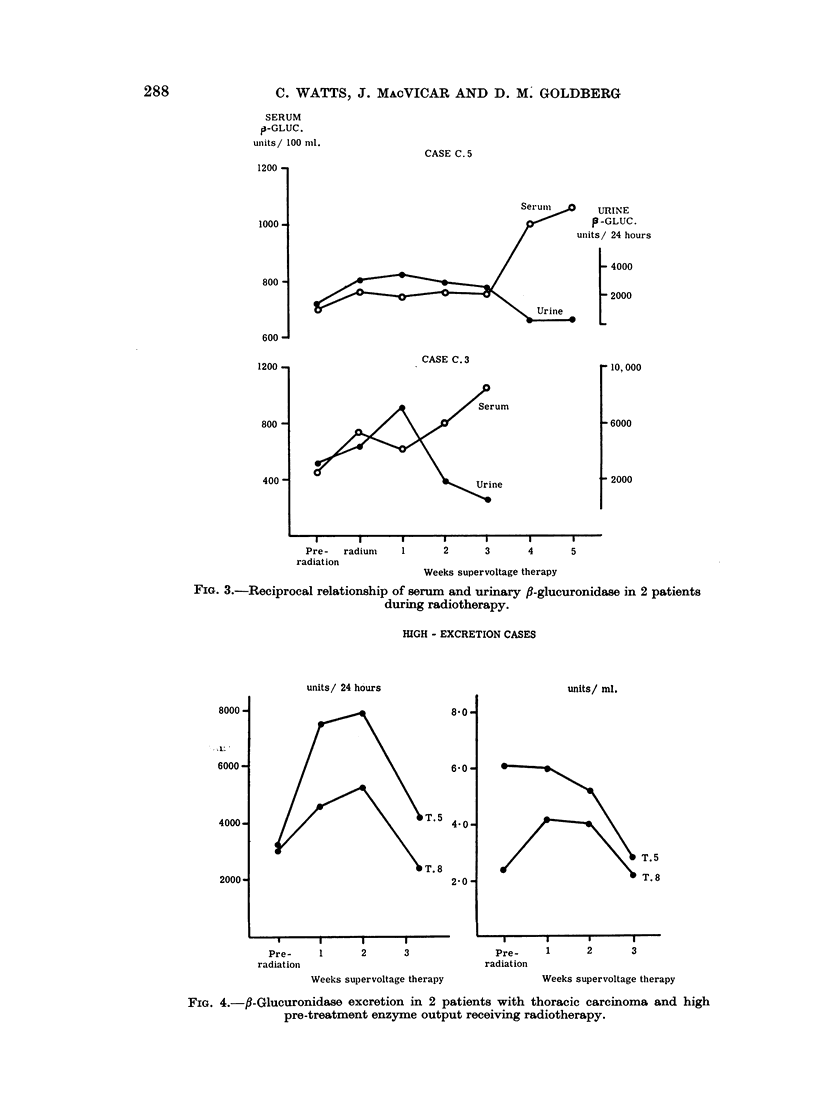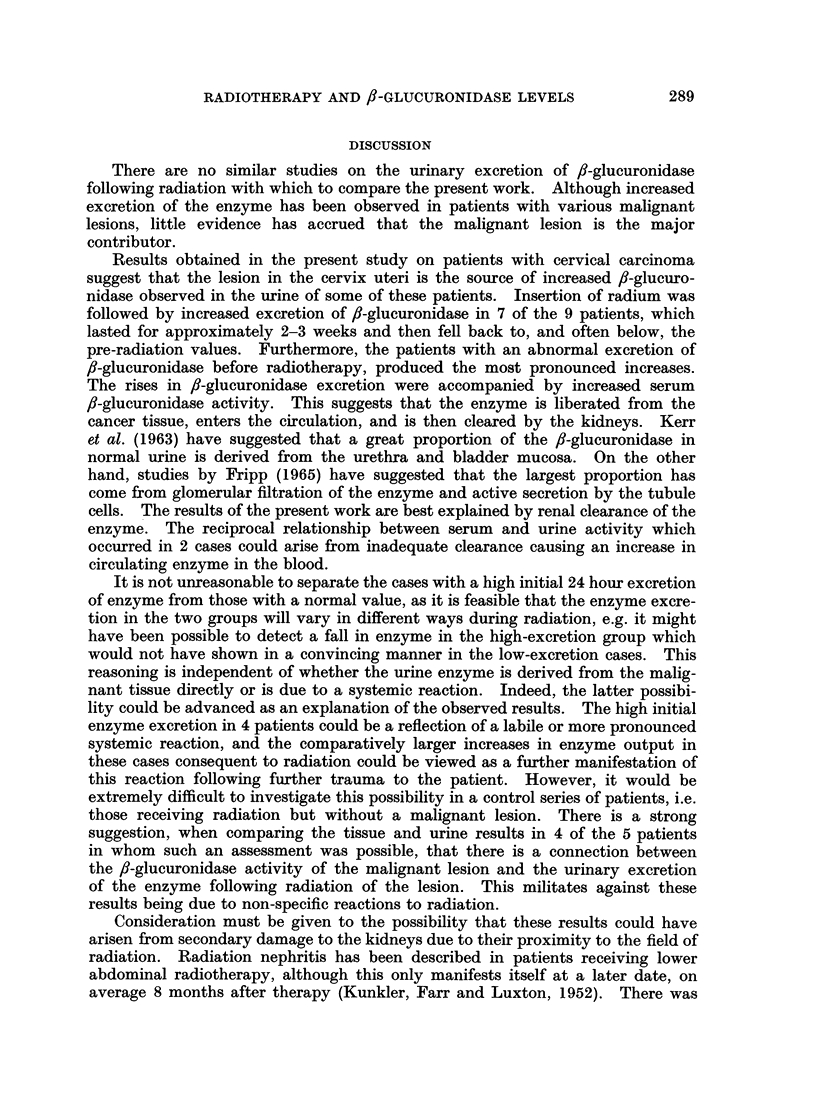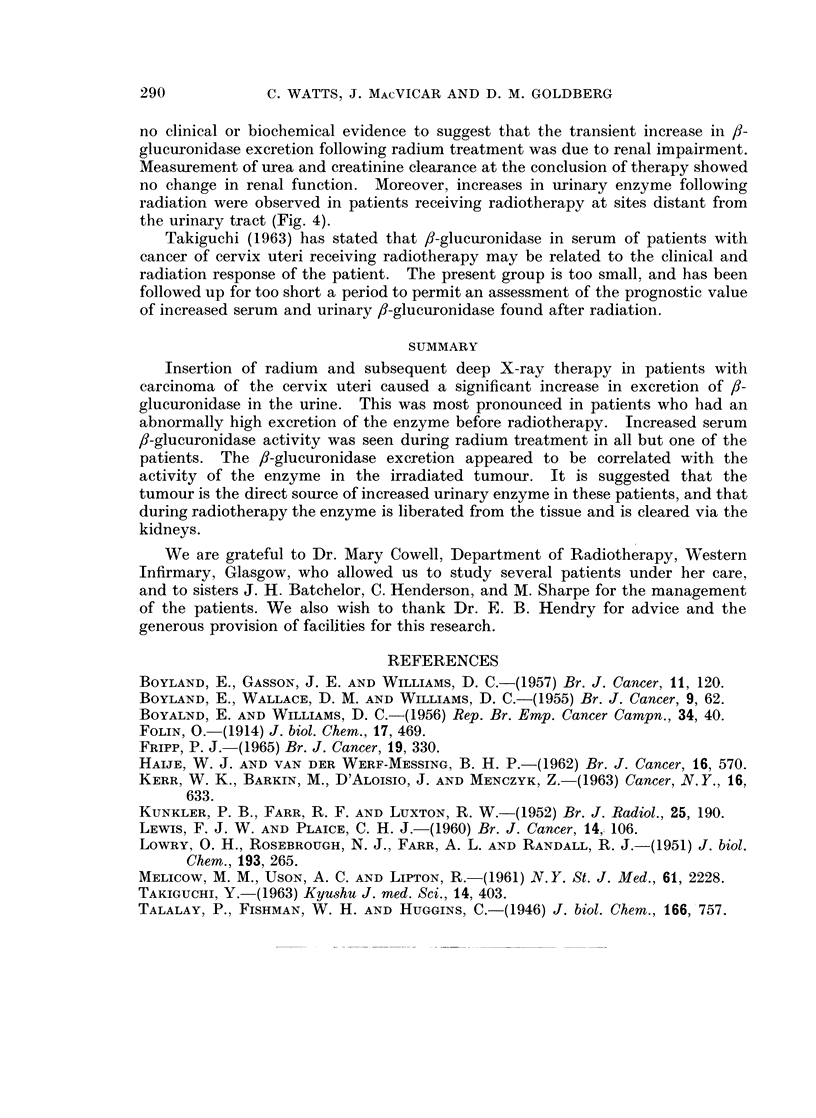# The effect of therapeutic radiation on serum and urine beta-glucuronidase.

**DOI:** 10.1038/bjc.1966.36

**Published:** 1966-06

**Authors:** C. Watts, J. MacVicar, D. M. Goldberg


### THE EFFECT OF THERAPEUTIC RADIATION ON SERUM AND URINE β-GLUCURONIDASE

C. WATTS, J. MACVICAR AND D. M. GOLDBERG
*From the Department of Biochemistry, and University Department of Pathological Biochemistry, Western Infirmary, Glasgow, W.2.*

Received for publication February 18, 1966

β-GLUCURONIDASE is a hydrolytic enzyme which splits β-glucuronic acid from a large number of natural and synthetic β-glucosiduronic acids. The enzyme is widely distributed in human tissues and body fluids and has measurable activity in blood serum and urine. Increased excretion of β-glucuronidase has been detected in the urine of some patients with malignant lesions (Boyland, Gasson and Williams, 1957); cases of carcinoma of the larynx, bronchus, oesophagus, prostate and testis showed the largest increases. The excretion of the enzyme has been investigated most fully, however, in patients with bladder carcinoma, where increased values have been found (Boyland, Wallace and Williams, 1955; Lewis and Plaice, 1960; Melicow, Uson and Lipton, 1961; Haije and van der Werf-Messing, 1962; Kerr, Barkin, D'Aloisio and Menczyk, 1963). Although the urinary enzyme has been found to be increased in a wide variety of malignant lesions, there has been little evidence that the malignant lesion itself is the primary source of this increase. Kerr *et al.* (1963) have shown that surgical removal of bladder tumours resulted in a fall in urinary β-glucuronidase from previously high values to normal, and Haije and van der Werf-Messing (1962) found raised values in cases of active bladder carcinoma but did not find elevated values in a group of patients who, after radiotherapy, showed no evidence of active tumour at follow-up.

It is of interest to know if other malignant lesions outwith the urinary tract may contribute directly to increased β-glucuronidase in the urine. Moreover, it is possible that increases might arise from a systemic reaction to the carcinoma, as the urinary activity can be raised during systemic reactions such as pyrexia (Boyland and Williams, 1956). There is also doubt whether the enzyme in the urine is derived from renal or post-renal sources. Kerr *et al.* (1963) regarded it as arising primarily from the ureters and bladder wall, but recently Fripp (1965) has suggested that the greater part derives from glomerular filtration and active secretion by the renal tubule cells.

In the present study, the excretion of β-glucuronidase in the urine of patients with carcinoma of the cervix uteri receiving radiotherapy was investigated. Evidence was obtained that the cervical lesion was presumably the primary source of increased β-glucuronidase in the urine of these patients, and that radiation of the lesion resulted in liberation of the enzyme from the tissue.

### MATERIAL AND METHODS

Patients with cancer of the cervix uteri, about to undergo a full course of radiotherapy, were selected for this study. All subjects could readily co-operate in the collection of specimens, were not incontinent of urine, showed no signs of urinary infection, and had good renal function at the time of presentation as measured by urea and creatinine clearance. They received total radiation doses ranging from 5000–8500 rads. These were given in part as local radium inserted for periods of 48–100 hours usually as two insertions, followed by a course of supervoltage therapy from the A.E.I. Linear Accelerator administered over a 3–5 week period.

Twenty-four hour collections of urine were taken periodically, using 10 ml. of chloroform as preservative. Two were collected before commencement of treatment, one during each period of radium insertion, one immediately following each insertion, and 2–3 times per week during the course of supervoltage therapy. Blood samples were also taken on days corresponding to the urine collections.

In 5 of the 9 patients in this series a biopsy of the malignant cervix was obtained just before insertion of the first radium implant. After removal, the tissue was placed in ice-cold 0·25 M-sucrose, cleansed of blood clots and exudate, dried and stored at −20° C. It was later homogenised in 0·25 M-sucrose and the supernatant obtained by centrifuging at 35,000 *g* for 60 minutes.

*Estimation of β-glucuronidase in urine, serum and tissue supernatant*

The pH of the urine was recorded, the volume accurately measured, and an aliquot of the well-mixed specimen was centrifuged at 2000 *g* for 10 minutes. The clear supernatant urine was stored at 4° C. unti lanalysis. The activity of β-glucuronidase in urine was measured by the method described by Melicow *et al.* (1961). A unit of enzyme activity is defined as that amount of enzyme which liberates 1 μg. phenolphthalein/hour at 37° C. under the test conditions. Urine β-glucuronidase activity is expressed as units/ml. urine or units excreted/24 hours. Urinary creatinine was also measured by the method of Folin (1914).

The activity of the enzyme in blood serum and tissue supernatant was measured by slight modification of the method of Talalay, Fishman and Huggins (1946). 0·1 ml. of test material was added to 0·8 ml. of 0·1 M-acetate buffer, pH 4·5, and 0·1 ml. of 0·05 M-phenolphthalein mono-β-glucuronic acid substrate. The mixture was incubated for 24 hours (serum), or periods of 1–4 hours (supernatant). Four ml. of 0·4 M-glycine buffer, pH 10·4, was then added, the tube centrifuged at 2000 *g* for 5 minutes, and the optical density of the clear supernatant read against a blank at 550 mμ. Standards of pure phenolphthalein were used to construct a calibration curve. The enzyme unit was the same as that defined for urine. The serum activity was expressed as units/100 ml. The protein content of the tissue supernatant was measured by the method of Lowry, Rosebrough, Farr and Randall (1951), and enzyme activity expressed as units/mg. protein.

### RESULTS

A normal range for urine β-glucuronidase was obtained by taking 24 hour urine collections from 20 healthy subjects aged 19–67 years. These gave a mean excretion of 1520 units/24 hours, S.D. 410, with an observed range of 950–2450, and a mean urine enzyme activity of 1·29 units/ml., S.D. 0·36, with an observed range of 0·74–1·93 units/ml. A normal range for serum β-glucuronidase from 30 healthy subjects aged 19–60 was found to be 200–1000 units/100 ml.

In order to group the results on all 9 patients together for statistical analysis, the scheme of radiotherapy for each patient was divided into phases, and the results calculated from urine collections and blood specimens taken during each phase. These were as follows :

1. *Pre-treatment*—comprising 2 consecutive 24 hour urine collections, with 2 blood specimens, taken before treatment commenced.

2. *Radium treatment*—five of the patients had 2 separate insertions of radium. Consequently, each had a total of 4 urine collections and blood specimens taken during radium treatment—2 with radium *in situ* and 2 following withdrawal. The remaining 4 patients had only one insertion and, therefore, only a total of 2 urine and blood specimens per patient.

3. *Supervoltage therapy*—this was divided into 3 separate weekly periods on all the patients. Some had a 4th or 5th week of therapy, but data from urine collections and blood specimens taken after the 3rd week have only been presented for 2 patients where they were of special significance.

The mean $\beta$-glucuronidase value for each patient at each phase of therapy was calculated for blood and urine from the results of all specimens taken during each phase. In the figures presenting data on the individual patients, each point will be a mean result from 2 to 4 separate specimens. In the tables of statistical data on the 9 patients as a group, the mean $\pm$ S.E. for each phase of treatment was calculated from the mean $\beta$-glucuronidase value of each individual patient.

The collective data on the 9 patients are shown in Table I, with the results

TABLE I.—*Activity of $\beta$-Glucuronidase in Urine during Radiotherapy*

| | Urine $\beta$-glucuronidase | | | |
|---|---|---|---|---|
| | Daily excretion units/24 hours | | Activity units/ml. urine | |
| | Mean $\pm$ S.E. | *P* | Mean $\pm$ S.E. | *P* |
| Pre-treatment | $2270 \pm 405$ | — | $1\cdot66 \pm 0\cdot25$ | — |
| Radium treatment | $3577 \pm 685$ | $< 0\cdot01$ | $2\cdot71 \pm 0\cdot45$ | $< 0\cdot01$ |
| Supervoltage | | | | |
| Week 1 | $4310 \pm 1023$ | $< 0\cdot02$ | $2\cdot84 \pm 0\cdot51$ | $< 0\cdot05$ |
| Week 2 | $3360 \pm 1133$ | n.s. | $2\cdot33 \pm 0\cdot48$ | n.s. |
| Week 3 | $3380 \pm 1436$ | n.s. | $1\cdot86 \pm 0\cdot38$ | n.s. |

Number of patients = 9
S.E. = standard error of the mean ; n.s. = not significant.
*P* values were obtained by applying the Student *t* test to the difference between the mean for each treatment and the pre-treatment value.

expressed as 24 hour excretion of enzyme and as units/ml. urine. They were also calculated as units/mg. urinary creatinine but this did not produce significant variation from those presented and so have not been included. As can be seen from the S.E. of the means during each treatment, there was a large patient variation in enzyme output before and after radiotherapy. In spite of this, it was shown statistically that an increase in urinary enzyme occurred during and following radium therapy. The concomitant increase in enzyme activity in units/ml. indicated a true increase in enzyme output and not simply increased urine volume.

There was, however, variation between the patients in the group. This is shown in Fig. 1. The cases were divided into those with an abnormally high pre-treatment $\beta$-glucuronidase excretion and those with normal values. It

FIG. 1.—Urinary excretion of $\beta$-glucuronidase during radiotherapy of carcinoma of cervix uteri.

should be noted that the largest increases in enzyme excretion occurred in those patients with high pre-treatment excretion. The very large increases seen in Case C.2 were accompanied by a diuresis occurring in the 2nd and 3rd weeks of supervoltage therapy ; there was, however, an increase also in enzyme activity as units/ml. in the 3rd week. Collections taken on this patient for a further 2 weeks of radiotherapy showed a fall in enzyme excretion over this period to approximately 7000 units/24 hours.

Of the 5 patients with normal pre-treatment $\beta$-glucuronidase excretion, 2—Cases C.5 and C.6—showed a definite increase in enzyme excretion occurring over the same period as the 4 high-excretion cases. Case C.7 appeared also to show a slight increase, although not to values outside the normal range. The remaining 2 cases showed no response to radiation and produced remarkably constant urine values over the whole course of therapy.

There appeared to be only a slight correlation between the degree of malignancy of the tumour and the $\beta$-glucuronidase response. The 4 patients in the high-excretion group all showed highly malignant features on histological examination, with poor differentiation and a high mitotic rate. However, in the low-excretion group Case C.8 which produced no response in urinary $\beta$-glucuronidase was classed similarly to these patients, while Cases C.5 and C.6, which showed the largest response in this group, had well-differentiated features with a low mitotic rate. The pattern of $\beta$-glucuronidase excretion was also not related to the total radiation dose administered.

Serum $\beta$-glucuronidase results from specimens taken at times corresponding to urine collections are shown in Table II. All except one case showed an increase

TABLE II.—*Activity of Serum $\beta$-Glucuronidase during Radiotherapy*

| | Serum $\beta$-glucuronidase (units/100 ml.) | |
|---|---|---|
| | Mean $\pm$ S.E. | $P$ |
| Pre-treatment . . . | $646 \pm 58$ | — |
| Radium treatment . . | $794 \pm 43$ | $<0 \cdot 01$ |
| Supervoltage treatment | | |
| Week 1 . . . | $662 \pm 73$ | n.s. |
| Week 2 . . . | $701 \pm 84$ | n.s. |
| Week 3 . . . | $676 \pm 85$ | n.s. |

in serum enzyme activity during radium therapy. Thereafter, results were unpredictable. This is shown in Fig. 2 where it can be seen that 3 cases showed further increases to abnormally high values during supervoltage therapy, 4 showed subsequent decreases, and 2 showed no obvious change.

It is clear that cases which produced increased excretion of $\beta$-glucuronidase during radium treatment also showed increased enzyme activity in the serum. Case C.8, which did not produce an increase in urine activity, also failed to show a rise in serum $\beta$-glucuronidase. There did not appear to be a connection between the urine and serum results over the period of supervoltage therapy, except in 2 cases. These are shown in Fig. 3 and appear to exhibit a reciprocal relationship between serum and urine $\beta$-glucuronidase. Both cases showed a coincident increase in urine and serum enzyme during radium therapy. Case C.5 then produced a dramatic fall in urine enzyme output to subnormal values of 200–400 units/24 hours during the 4th and 5th weeks of deep X-ray therapy, which was accompanied by a rise in serum enzyme activity to abnormal values.

Biopsy material for the analysis of tissue $\beta$-glucuronidase activity was obtained in Cases C.1, 2, 3, 5, and 8. Of the cases with a high initial excretion of the enzyme, C.2 and C.3 had a very high tissue activity—44 and 46 units/mg. protein (12 specimens of non-cancerous cervical tissue gave a range of 0·6–8 units/mg. protein). In the cases with normal pre-treatment excretion, C.5 had a moderate tissue activity—17 units/mg. protein—and showed an increase in urinary $\beta$-glucuronidase following radiation, although not so marked as Cases C.2 and C.3 ; Case C.8 showed no change in urine enzyme and had low tissue activity—7 units/mg. protein. From these results, there would appear to be a relationship between excretion of the enzyme in the urine following irradiation of the tissue by radium insertion and the β-glucuronidase activity of the carcinoma tissue. However, Case C.1 does not appear to uphold this hypothesis. This case had a high initial excretion of β-glucuronidase which increased following radium therapy, but the corresponding tissue specimen showed only a low enzyme activity of 6 units/mg. protein. It is possible that in this case the biopsy obtained was not truly representative of the tumour. The patient was found to have a highly

FIG. 2.—Serum β-glucuronidase during radiotherapy. Cases are grouped according to the pattern of enzyme response during supervoltage therapy.

malignant lesion, whereas the tissue β-glucuronidase activity was much lower than normally encountered in this type, the activity being in the non-cancerous range quoted above.

Fig. 4 shows data on 2 cases, one with carcinoma of the oesophagus and one with carcinoma of the bronchus, who had a high pre-treatment excretion of urinary β-glucuronidase and showed a pronounced increase in enzyme output during supervoltage therapy. The data were derived in the same way as the cervical carcinoma cases. These were only 2 cases in a group of 8 patients with carcinoma in the thoracic region who had a high initial excretion of β-glucuronidase. They have been included to illustrate that an increase in β-glucuronidase excretion can occur in patients receiving radiation distant from the renal tract.

FIG. 3.—Reciprocal relationship of serum and urinary β-glucuronidase in 2 patients during radiotherapy.

FIG. 4.—β-Glucuronidase excretion in 2 patients with thoracic carcinoma and high pre-treatment enzyme output receiving radiotherapy.

### DISCUSSION

There are no similar studies on the urinary excretion of $\beta$-glucuronidase following radiation with which to compare the present work. Although increased excretion of the enzyme has been observed in patients with various malignant lesions, little evidence has accrued that the malignant lesion is the major contributor.

Results obtained in the present study on patients with cervical carcinoma suggest that the lesion in the cervix uteri is the source of increased $\beta$-glucuronidase observed in the urine of some of these patients. Insertion of radium was followed by increased excretion of $\beta$-glucuronidase in 7 of the 9 patients, which lasted for approximately 2–3 weeks and then fell back to, and often below, the pre-radiation values. Furthermore, the patients with an abnormal excretion of $\beta$-glucuronidase before radiotherapy, produced the most pronounced increases. The rises in $\beta$-glucuronidase excretion were accompanied by increased serum $\beta$-glucuronidase activity. This suggests that the enzyme is liberated from the cancer tissue, enters the circulation, and is then cleared by the kidneys. Kerr *et al.* (1963) have suggested that a great proportion of the $\beta$-glucuronidase in normal urine is derived from the urethra and bladder mucosa. On the other hand, studies by Fripp (1965) have suggested that the largest proportion has come from glomerular filtration of the enzyme and active secretion by the tubule cells. The results of the present work are best explained by renal clearance of the enzyme. The reciprocal relationship between serum and urine activity which occurred in 2 cases could arise from inadequate clearance causing an increase in circulating enzyme in the blood.

It is not unreasonable to separate the cases with a high initial 24 hour excretion of enzyme from those with a normal value, as it is feasible that the enzyme excretion in the two groups will vary in different ways during radiation, e.g. it might have been possible to detect a fall in enzyme in the high-excretion group which would not have shown in a convincing manner in the low-excretion cases. This reasoning is independent of whether the urine enzyme is derived from the malignant tissue directly or is due to a systemic reaction. Indeed, the latter possibility could be advanced as an explanation of the observed results. The high initial enzyme excretion in 4 patients could be a reflection of a labile or more pronounced systemic reaction, and the comparatively larger increases in enzyme output in these cases consequent to radiation could be viewed as a further manifestation of this reaction following further trauma to the patient. However, it would be extremely difficult to investigate this possibility in a control series of patients, i.e. those receiving radiation but without a malignant lesion. There is a strong suggestion, when comparing the tissue and urine results in 4 of the 5 patients in whom such an assessment was possible, that there is a connection between the $\beta$-glucuronidase activity of the malignant lesion and the urinary excretion of the enzyme following radiation of the lesion. This militates against these results being due to non-specific reactions to radiation.

Consideration must be given to the possibility that these results could have arisen from secondary damage to the kidneys due to their proximity to the field of radiation. Radiation nephritis has been described in patients receiving lower abdominal radiotherapy, although this only manifests itself at a later date, on average 8 months after therapy (Kunkler, Farr and Luxton, 1952). There was no clinical or biochemical evidence to suggest that the transient increase in $\beta$-glucuronidase excretion following radium treatment was due to renal impairment. Measurement of urea and creatinine clearance at the conclusion of therapy showed no change in renal function. Moreover, increases in urinary enzyme following radiation were observed in patients receiving radiotherapy at sites distant from the urinary tract (Fig. 4).

Takiguchi (1963) has stated that $\beta$-glucuronidase in serum of patients with cancer of cervix uteri receiving radiotherapy may be related to the clinical and radiation response of the patient. The present group is too small, and has been followed up for too short a period to permit an assessment of the prognostic value of increased serum and urinary $\beta$-glucuronidase found after radiation.

SUMMARY

Insertion of radium and subsequent deep X-ray therapy in patients with carcinoma of the cervix uteri caused a significant increase in excretion of $\beta$-glucuronidase in the urine. This was most pronounced in patients who had an abnormally high excretion of the enzyme before radiotherapy. Increased serum $\beta$-glucuronidase activity was seen during radium treatment in all but one of the patients. The $\beta$-glucuronidase excretion appeared to be correlated with the activity of the enzyme in the irradiated tumour. It is suggested that the tumour is the direct source of increased urinary enzyme in these patients, and that during radiotherapy the enzyme is liberated from the tissue and is cleared via the kidneys.

We are grateful to Dr. Mary Cowell, Department of Radiotherapy, Western Infirmary, Glasgow, who allowed us to study several patients under her care, and to sisters J. H. Batchelor, C. Henderson, and M. Sharpe for the management of the patients. We also wish to thank Dr. E. B. Hendry for advice and the generous provision of facilities for this research.

### REFERENCES

Boyland, E., Gasson, J. E. and Williams, D. C.—(1957) *Br. J. Cancer*, **11**, 120.
Boyland, E., Wallace, D. M. and Williams, D. C.—(1955) *Br. J. Cancer*, **9**, 62.
Boyalnd, E. and Williams, D. C.—(1956) *Rep. Br. Emp. Cancer Campn.*, **34**, 40.
Folin, O.—(1914) *J. biol. Chem.*, **17**, 469.
Fripp, P. J.—(1965) *Br. J. Cancer*, **19**, 330.
Haije, W. J. and van der Werf-Messing, B. H. P.—(1962) *Br. J. Cancer*, **16**, 570.
Kerr, W. K., Barkin, M., D'Aloisio, J. and Menczyk, Z.—(1963) *Cancer, N.Y.*, **16**, 633.
Kunkler, P. B., Farr, R. F. and Luxton, R. W.—(1952) *Br. J. Radiol.*, **25**, 190.
Lewis, F. J. W. and Plaice, C. H. J.—(1960) *Br. J. Cancer*, **14**, 106.
Lowry, O. H., Rosebrough, N. J., Farr, A. L. and Randall, R. J.—(1951) *J. biol. Chem.*, **193**, 265.
Melicow, M. M., Uson, A. C. and Lipton, R.—(1961) *N.Y. St. J. Med.*, **61**, 2228.
Takiguchi, Y.—(1963) *Kyushu J. med. Sci.*, **14**, 403.
Talalay, P., Fishman, W. H. and Huggins, C.—(1946) *J. biol. Chem.*, **166**, 757.